# The use of methylene blue in adult patients with septic shock: a systematic review and meta-analysis

**DOI:** 10.1016/j.bjane.2024.844580

**Published:** 2024-11-29

**Authors:** Ka Ting Ng, Pei En Kwok, Wei En Lim, Wan Yi Teoh, Mohd Shahnaz Hasan, Mohd Fitry Zainal Abidin

**Affiliations:** a University of Malaya, Department of Anesthesiology, Kuala Lumpur, Malaysia; b Quinnipiac University Frank H. Netter School of Medicine, Waterbury Hospital, Department of Surgery, North Haven, United States; c University of Glasgow, Department of Anesthesiology, Glasgow, United Kingdom

**Keywords:** Methylene blue, Septic shock, Nitric oxide, Lactate levels, Mortality, Systematic review

## Abstract

**Objectives:**

Methylene blue exerts its vasopressor properties by inhibiting nitric oxide-mediated vasodilation. Recent studies have advocated the use of methylene blue as a rescue therapy for patients with septic shock. The primary aim was to investigate the effect of methylene blue on the mean arterial pressure among adult patients with septic shock.

**Methods:**

Databases of MEDLINE, EMBASE, and CENTRAL were searched from their inception date until October 2023. Randomized Clinical Trials (RCT) comparing methylene blue and placebo in adults with septic shock were included.

**Results:**

Our systematic review included 5 studies (n = 257) for data analysis. As compared to the placebo, our pooled analysis showed that methylene blue significantly increased mean arterial pressure (MD: 1.34 mmHg, 95% CI 0.15 to 2.53, p = 0.03, level of evidence: very low). Patients who were given methylene blue were associated with statistically lower mortality rate (OR = 0.49, 95% CI 0.27 to 0.88, p = 0.02, level of evidence: low), reduced serum lactate levels (MD: -0.76 mmoL.L^-1^, 95% CI -1.22 to -0.31, p = 0.0009, level of evidence: low), reduced length of hospital stay (MD: -1.94 days, 95% CI -3.79 to -0.08, p = 0.04, level of evidence: low), and increased PaO_2_/FiO_2_ (MD: 34.78, 95% CI 8.94 to 60.61, p = 0.008, level of evidence: low).

**Conclusions:**

This meta-analysis demonstrated that methylene blue administration was associated with an increased in mean arterial pressure and PaO_2_/FiO_2_ ratio, along with a reduction in mortality rates, serum lactate levels, and length of hospital stay. However, substantial degree of heterogeneity and inadequate number of studies with low level of evidence warrant future adequately powered RCTs to affirm our results.

## Introduction

Sepsis is a life-threatening condition, resulting from a dysregulated host response to infection.^1^ Septic shock is characterized by persistent hypotension and elevated lactate levels (> 2 mmoL.L^-1^) that are unresponsive to fluid resuscitation.^1^ It is classified as a vasodilatory shock, marked by reduced myocardial contractility, widespread vasodilatation, and diminished responsiveness to vasopressors.^2^ Research has identified three primary mechanisms underlying vasopressor-resistant septic shock: vasopressin deficiency, activation of Adenosine Triphosphate (ATP)-sensitive potassium channels, and increased nitric oxide synthesis in vascular smooth muscle cells.^2^

According to the 2021 Surviving Sepsis Campaign guidelines, norepinephrine, epinephrine, or dopamine, and vasopressin are recommended as first-line, second-line and third-line therapies for treating septic shock, respectively.^3^ Prolonged stimulation of adrenergic receptors by norepinephrine, epinephrine, and dopamine may result in receptor downregulation and desensitization, leading to diminished responsiveness to vasopressor therapy.^4^ Additionally, studies have demonstrated that the use of catecholamines, dopamine, and vasopressin in septic shock can cause various adverse effects, including digital ischemia, mesenteric ischemia, and an increased incidence of arrhythmic events, respectively.^5^^,^^6^ Given the risks of adverse effects associated with these vasopressor agents, there is a growing interest in vasopressor-sparing strategies. Non-adrenergic adjuncts, such as methylene blue are being explored as alternatives to increase Mean Arterial Pressure (MAP) and maintain organ perfusion.

Nitric Oxide Synthase (NOS) produces Nitric Oxide (NO) from its derivative L-arginine, which activates a second messenger (guanylate cyclase and cyclic guanosine monophosphate) to cause vasodilatation.^7^ Nitric oxide synthase can be subdivided into two types, Namely Constitutive (cNOS) that is constantly active, also known as endothelial NOS (eNOS) and Inducible (iNOS) that is produced in large quantities upon activation by cytokines, endotoxin, and other inflammatory mediators.^7^ Nitric oxide has both beneficial and harmful effects in septic shock. While it reduces vascular responsiveness to catecholamines, it concurrently enhances oxygen delivery, promotes free radical scavenging, and stimulates macrophage activity.^8^ Given its varied impact, non-selective blockade of NOS inhibitors can be detrimental. A phase III clinical trial by Lopez et al. demonstrated that non-selective NOS inhibitors increased mortality and morbidity, including cardiac arrest and pulmonary hypertension, in patients with septic shock.^9^ This finding underscores the importance of preserving the function of the constitutive isoform (cNOS) to maintain homeostasis, even in the context of sepsis. These results suggests that a more targeted approach to modulating nitric oxide synthase and its downstream pathways is warranted.

Methylene blue is one of the earliest synthetic drugs to be used in medicine, which was first employed to treat patients with septic shock in the 1990s.^10^ It exhibits the potential of targeted inhibition on the iNOS, which demonstrated favourable hemodynamic effects with increased MAP and cardiac function.^11^^,^^12^ Experimental studies showed that methylene blue normalized plasma concentrations of nitric oxide end-products, improved cardiopulmonary function, and reversed endotoxin-induced hypotension in animals with endotoxemia.^13^^,^^14^ However, the inhibition of iNOS is associated with side effects on cardiovascular parameters and gas exchange.^15^

Current findings on the effects of methylene blue yielded mixed results. In a systematic literature review published in 2006, which included 14 studies, Kwok et al. highlighted that methylene blue increased systemic vascular resistance and MAP.^16^ However, Porizka et al. showed that there are no significant differences between methylene blue administration and MAP among patients with refractory distributive shock.^17^ In addition, studies have administered methylene blue in doses ranging from 1 mg.kg^-1^ to 7 mg.kg^-1^.^16^^,^^18^ Some studies employed bolus-only administration, while others utilized continuous infusion or a combination of bolus infusion followed by continuous infusion.^16^^,^^19^ Consequently, there is no clear consensus on the optimal dosing strategy for administering methylene blue. To address this knowledge gap, a systematic review and meta-analysis assessing the efficacy and safety profile of methylene blue is timely and essential before making any recommendation for its use in patients with septic shock.

We hypothesized that methylene blue administration improves MAP in patients with septic shock. The primary objective of this systematic review and meta-analysis was to evaluate the clinical efficacy of methylene blue in improving MAP in septic shock patients. Secondary objectives included examining its effects on mortality rate, serum lactate levels, length of hospital stay, heart rate, and the ratio of arterial oxygen partial pressure to fractional inspired oxygen (PaO_2_/FiO_2_).

## Methods

This review was conducted according to the guidelines outlined by the Cochrane Handbook for Systematic Reviews and Interventions.^20^ Our protocol was registered and published in the database of PROSPERO under CRD42023460671 prior to commencement of literature search. Review questions were formed based on the Population (adult patients with septic shock), Intervention (Methylene Blue), Comparison (control drugs), and Outcomes framework. The primary aim of the review is to examine the effect of methylene blue on MAP. Other secondary aims include mortality rate (all-cause mortality rate for the longest follow-up), serum lactate, length of hospital stay, heart rate and PaO_2_/FiO_2_.

### Literature search and study identification

Databases of MEDLINE, EMBASE and the Cochrane Central Register of Controlled Trials (CENTRAL) were searched from their inception until October 2023. Clinical trial registries were searched systematically for ongoing trials. The definition of septic shock was tabulated in Supplementary Table 1 whereas the search strategies and terms utilized in this review was shown in the Supplementary Table 2. In general, eligibility criteria were: 1) All Randomized Controlled Trials (RCT) comparing methylene blue versus control group were included. 2) All RCTs comparing methylene blue versus control group involving adults (> 18-years-old) with septic shock, regardless of reported outcomes were included.

No language restrictions were applied. From our search, we included non-English studies, in which these articles were translated to English with the help of certified translators. Observational studies, case series or reports, conference abstract were excluded. The references of the included RCTs were manually searched and cross referenced for further potential studies. Authors of relevant studies were contacted at least three times for any incomplete data.

### Study selection and data extraction

The review was reported in accordance with the guideline from the Preferred Reporting Items for Systematic Reviews and Meta-Analyses Statement 2020.^21^^,^^22^ Two authors (PK and WL) briefed by the principal author (KN) for the inclusion and exclusion criteria. Titles and abstracts were screened for eligibility criteria by two authors (PK and WL) independently. The final selection of all the included RCTs were discussed and agreed among all the authors (KN, PK, WL). Any disagreements were solved by the principal author (KN). The clinical characteristics of all included studies were recorded independently by two authors (PK and WL) using an online data extraction sheet. Our data extraction sheet included the following important data: author, publication year, type of control drug, dosage of control drug and methylene blue, mean age of both intervention and control group, mean weight of both groups, timing of administration of methylene blue, and sample sizes.

### Risk of bias assessment

The included RCTs were assessed for their respective risk of bias according to the Cochrane Collaboration Risk of Bias Assessment Tool by 2 authors (PK and WL) independently.^23^ Any disagreements were resolved via discussions with the principal author (KN). Summary of findings and the assessment on the level of evidence were conducted by 2 authors (PK and WL) independently. These assessments were evaluated using the GRADEpro software.^24^ A third author (KN) was consulted to solve any conflicts.

### Statistical analysis

Review Manager version 5.4 (The Cochrane Collaboration, Copenhagen, Denmark) was used to carry out statistical meta-analysis.^25^ All p-values were two tailed, with statistical significance denoted as less than 0.05. With regards to dichotomous outcomes, Odd Ratios (OR) and 95% Confidence Interval (95% CI) were calculated. For continuous outcomes, Mean Difference (MD) and 95% Confidence Interval (95% CI) were utilized. The heterogeneity of pooled outcomes was evaluated by the I-square (I^2^) test, where the values of < 40%, 40%‒60% and > 60% were indicated as low, moderate, and high heterogeneity, respectively. A fixed-effect model was used to calculate the estimates of the primary and secondary outcomes. However, if high heterogeneity (I^2^ > 60%) was observed, a random-effect model was used. When the values were reported as median or interquartile range, these values were converted to mean and standard deviation.^26^ Publication bias was evaluated using funnel plots for any asymmetry.^27^ Subgroup analysis was performed on specific time intervals on the primary outcome of MAP. Two specific time-intervals, namely immediate period after administration of methylene blue and 3-day after methylene blue administration, were chosen to study its immediate effects and its effects after being eliminated from the body.^28^

## Results

**Figure 1:**
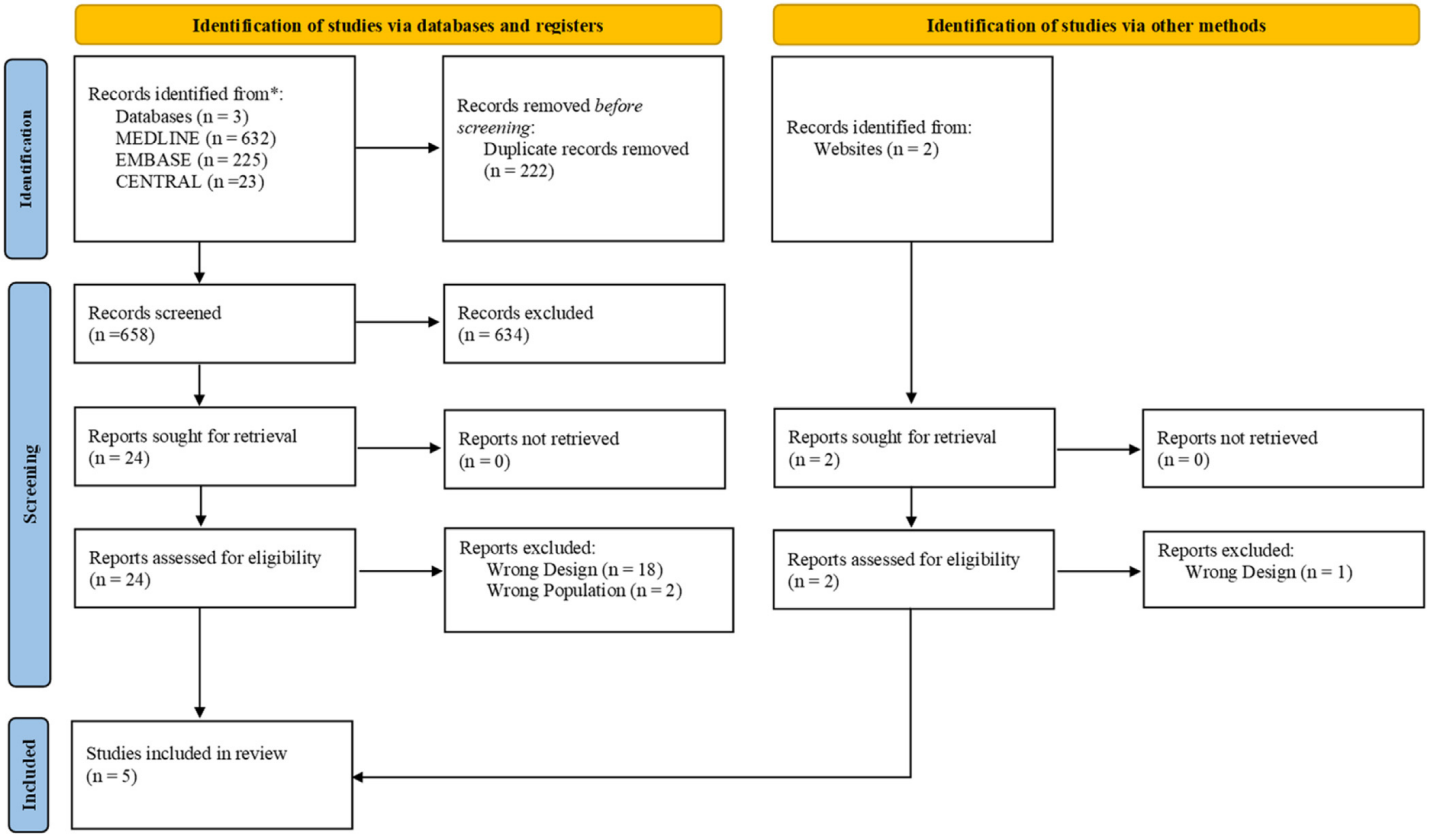
Prisma diagram. A total of 5 studies were included in this systematic review.

PRISMA flow chart of the study selection was illustrated in Figure 1. After removing duplicates, 658 articles remained for title and abstract screening. A total of 26 articles were downloaded for full text screening. A total of 21 papers were excluded due to failure to fulfil the inclusion criteria. The list of excluded studies is shown in Supplementary Table 3. Five articles with 257 participants were eligible to be included in this review. The demographics of the included RCTs are illustrated in Table 1. Searching of trial registries found 1 ongoing study, which is shown in Supplementary Table 4.

**Table 1:** Clinical characteristics of included studies.

Author, Year	Methylene blue dose regimes	Control Group	Sample Size	Main Findings
Kirov et al., 2001	Intravenous bolus injection (2 mg.kg^-1^) methylene blue, followed 2 hours later by infusion at stepwise increasing rates of 0.25, 0.5, 1, and 2 mg.kg^-1^.hour^-1^ that were maintained for 1 hour each	Normal Saline	20	Methylene blue reduced the requirement for norepinephrine, epinephrine, and dopamine by as much as 87%, 81%, and 40%, respectively.
				Mean arterial pressure was significantly increased at the 6-hour and 24-hour after drug administration in the methylene blue group compared to the control group.
Arzapalo et al., 2016	2 mg.kg^-1^ methylene blue in 100 mL of 5% glucose solution infused over 1 hour	Glucose	60	Methylene blue group (22%) was associated with an increase mean arterial pressure than the control group (9.2%).
				Noradrenaline dose at the 6-hour was significantly reduced in the methylene blue group (86%) as compared to the control group (56%).
Lu et al., 2018	2 mg.kg^-1^ methylene blue (diluted with 50 mL of 0.9% sodium chloride injection) into a single intravenous pump. After 20 minutes, continue administering methylene blue at 2 mL/hour for 24 hours	Normal Saline	32	Mean arterial pressure after day 1 of drug administration was significantly higher in the methylene blue group than the control group.
				Perfusion index at day 1 to day 5 after drug administration was significantly higher in the methylene blue group than the control group.
				Oxygenation index at day 2 to day 5 after drug administration was significantly higher in the methylene blue group than the control group.
Lu et al., 2019	Group 1: Bolus Infusion (2 mg.kg^-1^ of methylene blue added into 50 mL of normal saline infused over 20 mins, and then continuous infusion of 50 mL of normal saline over 24 hours)	Normal Saline	54	Norepinephrine dosage at day 1 was significantly lower in the methylene blue group than the control group.
	Group 2: Continuous Infusion (2 mg.kg^-1^ of methylene blue added into 50 mL of normal saline infused over 20 mins, and then continuous infusion of methylene blue diluted with 50 mL of normal saline over 24 hours)			Methylene Blue significantly increased perfusion index at day 1 to day 7 compared to the control group.
Ibarra-Estrada et al., 2023	100 mg of methylene blue in 500 mL of 0.9% sodium chloride solution over 6 hours once daily for a total of 3 doses	Normal Saline	91	The methylene blue group had a significantly shorter time to vasopressor discontinuation, a shorter ICU length of stay by 1.5 days and shorter hospital length of stay by 2.7 days as compared to the control group.

Data are presented as mean ± standard deviation. RCT, Randomised Controlled Trials.

The control drugs varied among the included studies. Three studies used normal saline as the control drug,^29^, ^30^, ^31^ whilst others employed glucose or alternative fluid resuscitation treatments.^32^ The range of mean age of the control group is 23.8 to 76.0 years old whereas the range of mean age of the methylene blue group is 33.8 to 75.0 years old, respectively. The mean body mass index of the control group is 22.2 to 28.4 kg.m^-2^ whereas the mean body mass index of the methylene blue group is 22.1 to 27.6 kg.m^-2^. Of all, the year of publication ranged from 2001 to 2023 with sample sizes of each group varied from 10 to 46. Data analysis of primary and secondary outcomes is illustrated in Table 2. Summary of findings and certainty of evidence is shown accordingly in Table 3.

**Table 2:** Data analysis of primary and secondary outcomes.

Outcomes	Trials	n	I^2^ (%)	MD/OR (95% CI)	p-value
Mean Arterial Pressure (Main Analysis) (mmHg)	6	286	99	1.34 [0.15, 2.53]	0.03
Mean Arterial Pressure (Subgroup Analysis) (mmHg)					
Time-points after MB Infusion					
a) Immediate period after MB Infusion	3	203	99	3.25 [0.24, 6.27]	0.03
b) 3-day after MB Infusion	3	183	29	0.22 [-0.30, 0.73]	0.42
Risk of Bias					
a) Low Risk of Bias	4	302	100	0.95 [-0.25, 2.15]	0.12
b) High/Unclear Risk of Bias	4	84	25	9.95 [3.51, 16.40]	0.002
Mortality Rate (Main Analysis)	4	259	0	0.49 [0.27, 0.88]	0.02
Mortality Rate (Subgroup Analysis)					
Time-points after MB Infusion					
a) 7-day	2	114	0	0.38 [0.13, 1.10]	0.08
b) 28-day	2	145	0	0.54 [0.26, 1.12]	0.10
Serum Lactate (Main Analysis) (mmoL.L^-1^)	6	413	45	-0.76 [-1.22, -0.31]	0.0009
Serum Lactate (Subgroup Analysis) (mmoL.L^-1^)					
Time-points after MB Infusion					
a) 1-hr after MB Infusion	2	77	0	0.00 [-0.94, 0.94]	1.00
b) 24-hr after MB Infusion	2	168	0	-1.06 [-1.32, -0.81]	<0.00001
c) 72-hr after MB Infusion	2	168	69	-1.04 [-2.26, 0.18]	0.09
d) PaO_2_/FiO_2_ (Main Analysis)	3	131	0	34.78 [8.94, 60.61]	0.008
PaO_2_/FiO_2._ (Subgroup Analysis)					
Time-points after MB Infusion					
a) Immediate Period After MB Infusion	2	111	0	34.37 [7.55, 61.19]	0.01
b) 24-hr after MB Infusion	1	20	N/A	40.00 [-56.51, 136.51]	0.42
Length of Hospital Stay (days)	2	111	0	-1.94 [-3.79, -0.08]	0.04
Heart Rate (Main Analysis) (bpm)	13	470	0	-2.31 [-5.88, 1.27]	0.21
Heart Rate (bpm)					
a) After MB Infusion	3	106	0	2.62 [-5.02, 10.25]	0.50
b) 24-hr after MB Infusion	3	106	10	-3.27 [-11.75, 5.22]	0.45
c) 72-hr after MB Infusion	2	86	0	-3.24 [-12.00, 5.52]	0.47
d) 5-day after MB Infusion	2	86	0	-3.26 [-11.34, 4.82]	0.43
e) 7-day after MB Infusion	3	86	0	-4.95 [-12.61, 2.70]	0.20

Values are Mean Difference (MD)/Odds Ratio (OR) and 95% Confidence Interval.

MB, Methylene Blue; PaO_2_/FiO_2_, Ratio of arterial oxygen partial pressure to fractional inspired oxygen; n, Sample Size; N/A, Not Applicable.

**Table 3:** Level of Evidence. Author(s): Ka Ting Ng, Pei En Kwok, Wei En Lim, Mohd Shahnaz Bin Hasan, Rafidah Atan, Nor’ Azim Bin Mohammed Yunus, Mohd Fitry Zainal Abidin. **Question:** Methylene Blue compared to Placebo for Septic Shock in Adults. **Setting:** Septic Shock Adult Patients.

Certainty assessment							N° of patients		Effect			
N° of studies	Study design	Risk of bias	Inconsistency	Indirectness	Imprecision	Other considerations	Methylene Blue	Placebo	Relative (95% CI)	Absolute (95%CI)	Certainty	Importance
**Mean Arterial Pressure (mmHg)**												
4	Randomized trials	Serious^a^	Serious^b^	Not serious	Serious^c^	None	142	144	‒	MD **2.94 higher** (1.52 higher to 4.36 higher)	⊕○○○ Very low	
**Mortality Rate**												
3	Randomized trials	Serious^a^	Not serious	Not serious	Serious^c^	None	27/147 (18.4%)	39/112 (34.8%)	**OR 0.49** (0.27 to 0.88)	**141 fewer per 1,000** (from 222 fewer to 28 fewer)	⊕⊕○○ Low	
**Serum Lactate (mmoL.L^-1^)**												
2	Randomized trials	Serious^a^	Not serious	Not serious	Serious^c^	None	228	185	‒	MD **0.76 lower** (1.22 lower to 0.31 lower)	⊕⊕○○ Low	
**PaO_2_/FiO_2_** (ratio of arterial oxygen partial pressure to fractional inspired oxygen)												
2	Randomized trials	Serious^a^	Not serious	Not serious	Serious^c^	None	65	66	‒	MD **34.78 higher** (8.94 higher to 60.61 higher)	⊕⊕○○ Low	
**Heart Rate (bpm)**												
3	Randomized trials	Serious^a^	Not serious	Not serious	Serious^c^	None	280	190	‒	MD **2.31 lower** (5.88 lower to 1.27 higher)	⊕⊕○○ Low	
**Length of Hospital Stay (days)**												
2	Randomized trials	Serious^a^	Not serious	Not serious	Serious^c^	None	55	56	‒	MD **1.94 lower** (3.79 lower to 0.08 lower)	⊕⊕○○ Low	

CI, Confidence Interval; MD, Mean Difference; OR, Odds Ratio.

Explanations:

a Half of the included studies possess high or unclear risk of bias.

b High degree of heterogeneity.

c Sample size of each arm < 400.

Overall, two of the included RCTs have a low risk of bias^30^^,^^32^ whereas the other 3 studies were evaluated as unclear or high risk of bias^29^, ^30^, ^31^ due to lack of blinding of participants and personnel, and lack of blinding of outcomes (Supplementary Table 5). The review was conducted with the aid of PRISMA checklist, as shown in Supplementary Table 6.

### Primary outcome: mean arterial pressure

**Figure 2:**
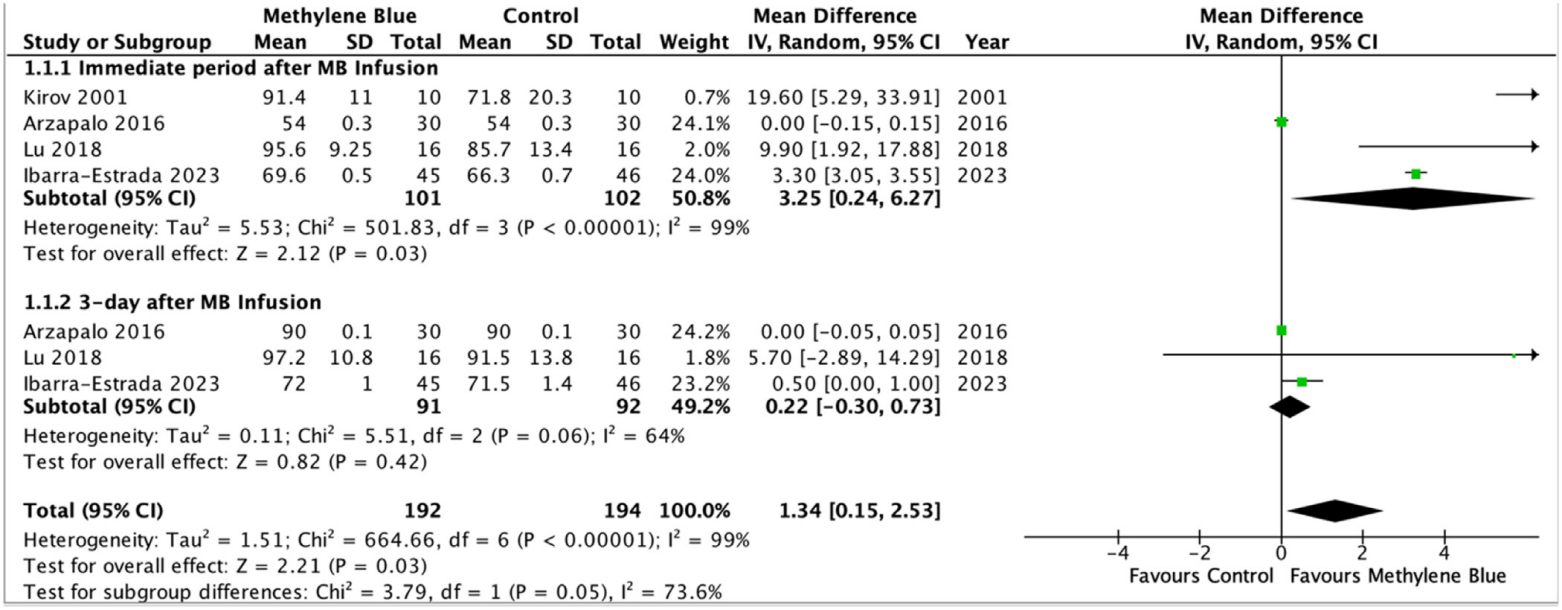
Forest plot of mean arterial pressure (mmHg) ‒ Methylene blue is associated with a significant increase in mean arterial pressure compared to the control group.

Three randomized-controlled trials examined the impact of methylene blue on MAP.^29^^,^^30^^,^^33^ Methylene blue significantly increased MAP in patients randomized to receiving methylene blue (n = 203, MD = 1.34 mmHg, 95% CI 0.15 to 2.53, p = 0.03, level of evidence: very low) (Figure 2). However, there is a high degree of heterogeneity for this measured outcome (I^2^ = 99%) and the funnel plot for MAP were asymmetrical, suggesting risk of publication bias. Subgroup analysis of specific time intervals revealed that methylene blue significantly increased MAP of patients in the immediate period after methylene blue infusion (studies = 4, n = 203, MD = 3.25, 95% CI 0.24 to 6.27, p = 0.03) (Supplementary Fig. 1).

### Secondary outcome: mortality rate, serum lactate, PaO_2_/FiO_2_, length of hospital stay, heart rate

**Figure 3:**
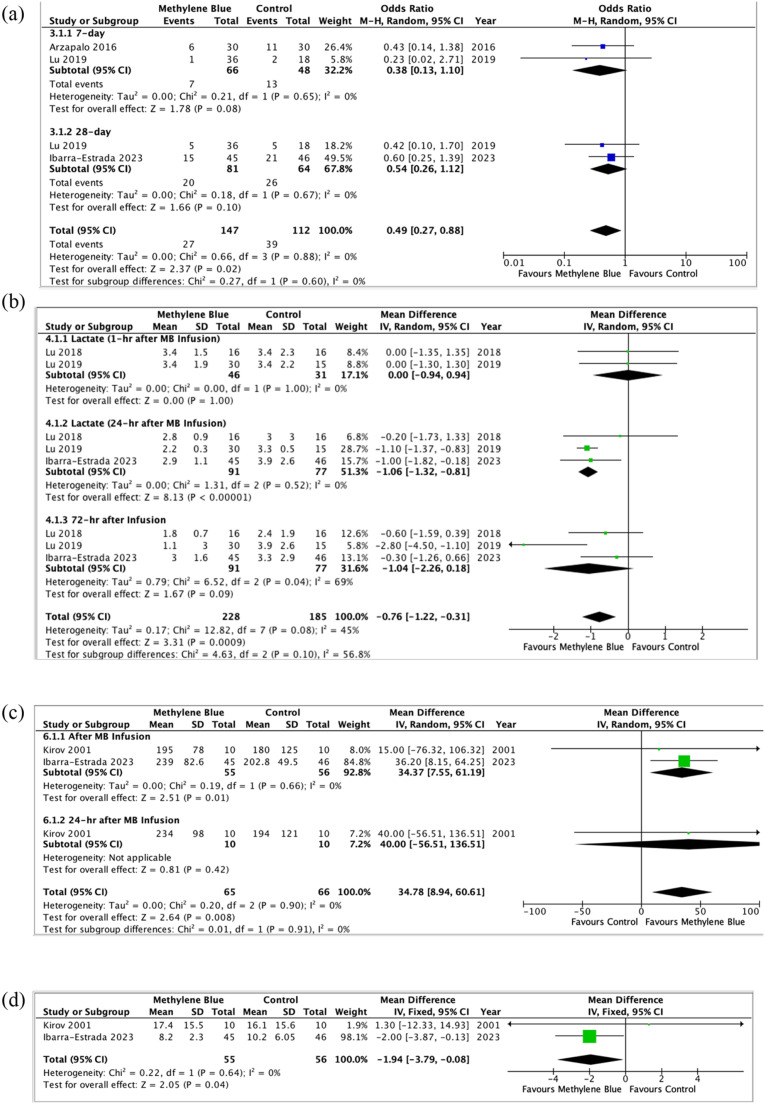
Comparison of mortality rate (a), serum lactate levels (b), PaO_2_/FiO_2_ ratio (c), and length of hospital stay (d), between the methylene blue group and the control group.

Based on the combined data from 3 RCTs, patients who received methylene blue had statistically lower mortality rate (n = 205, OR = 0.49, 95% CI 0.27 to 0.88, p = 0.02, level of evidence: low) (Figure 3).^30^, ^31^, ^32^ Low statistical heterogeneity was denoted across included studies (I^2^ = 0%). Pooled mean difference from 3 included studies showed that administration of methylene blue was associated with lower serum lactate levels compared to placebo (MD = -0.76 mmoL.L^-1^, 95% CI -1.22 to -0.31, p = 0.0009, level of evidence: low) (Figure 3).^30^^,^^31^^,^^33^ However, a moderate degree of heterogeneity was assessed in the pooled effect (I^2^ = 45%).

There was a significant increase in PaO_2_/FiO_2_ in the methylene blue group than the control group (MD = 34.78, 95% CI 8.94 to 60.61, p = 0.008, level of evidence: low) (Figure 3).^29^^,^^30^ A total of 2 studies (n = 111) measured the length of hospital stay. Patients who received methylene blue had a statistically shorter length of hospital stay as compared with the placebo group (MD = -1.94, 95% CI -3.79 to -0.88, p = 0.04, I^2^ = 0%, level of evidence: low) (Figure 3).^29^^,^^30^

Three studies (n = 106 patients) indicated showed that there was no significant difference between the methylene blue and the control group on the heart rate (MD = -2.31, 95% CI -5.88 to 1.27, p = 0.21, level of evidence: low) (Supplementary Fig. 2). The test for statistical heterogeneity was low (I^2^ = 0%).^29^, ^30^, ^31^

## Discussions

Our meta-analysis revealed that the methylene blue administration in patients with septic shock is associated with an increase in MAP over a short-term period following its use, although the results show considerable variability. Additionally, methylene blue is likely associated with improvement in the PaO_2_/FiO_2_ ratio, as well as reductions in mortality rate, serum lactate levels, and length of hospital stay.

A previous systematic review completed by Kwok et al. included 2 randomized-controlled trials and 11 case reports/series.^16^ Another systematic review by Paciullo et al. included 2 randomized-controlled trials and 8 prospective observational studies.^19^ However, the inclusion of non-RCTs would have downgraded the quality of evidence for the measured outcomes.^34^ Previously, no quantitative meta-analysis of the data could be performed due to the limited number of RCTs focusing on patients with septic shock. Given that more RCTs have been published, our team decided to perform an updated and comprehensive review and meta-analysis on the use of methylene blue in the adult population with septic shock.

Prompt treatment of hypotension is imperative in septic shock.^3^ Observational studies of methylene blue in septic shock patients have generally shown short term improvements in hemodynamic parameters.^12^^,^^15^^,^^16^^,^^35^ It is believed that methylene blue increases blood pressure via the inhibition of iNOS vasodilation, instead of vasoconstriction in catecholamines.^30^ Memis et al. reported that methylene blue statistically increased MAP in patients with severe sepsis.^36^ Similarly, our pooled analysis reported a statistical increase of MAP in the methylene blue group compared to the control group. However, the increase in MAP observed after 24 hours was not statistically significant in our findings. This suggests that the hemodynamic effects of methylene blue may be short-lived and transient. While methylene blue has long been established as the treatment of methemoglobinemia with dosages of 1‒2 mg.kg^-1^,^37^ the optimal dosing strategy for methylene blue in septic patients is still not well defined. A study on seven healthy individuals where 100 mg of IV methylene blue was administered demonstrated that the estimated terminal half-life is 5.25 hours.^38^ Three dosing strategies were identified in our study ‒ single bolus, continuous infusion, and bolus dose followed by infusion. Doses based on weight ranged from 0.5 mg.kg^-1^.h^-1^ to 2.0 mg.kg^-1^.h^-1^. The most commonly used regimen, observed in four out of five included studies, was continuous infusion.^29^^,^^31^, ^32^, ^33^ Ibarra-Estrada et al. was the only study to administer methylene blue for more than 24 hours, using repeated doses once daily for a total of three days.^30^ Juffermans et al. demonstrated that the hemodynamic effects of methylene blue are dose-dependent, with an infusion of 1‒3 mg.kg^-1^ being sufficient to increase MAP.^18^ This study also highlighted that the effects of a single infusion are transient, lasting approximately two hours.^18^ These findings align with our study, which showed a statistically significant increase in MAP immediately after methylene blue infusion but no significant effect 24 hours post-infusion. Therefore, repeated boluses or continuous infusion may be considered to mitigate this limitation. A significant degree of heterogeneity was observed in the primary outcome, likely due to factors such as variability in baseline patient characteristics, differing dosing regimens, timing of drug administration, and types of control drugs used. As such, future randomized controlled trials in a more homogenous clinical setting are necessary to accurately assess the impact of methylene blue on MAP in adult patients with septic shock.

In the setting of sepsis, inflammatory cytokines have been theorized to suppress cortisol response or compete with intracellular glucocorticoid receptors, resulting in peripheral tissue resistance and eventual vascular collapse.^39^^,^^40^ The utility of steroids was not explicitly described in all the included studies, except for Ibarra-Estrada et al.^30^ This study was the only RCT that administered intravenous hydrocortisone at 200 mg/day by continuous infusion as part of the resuscitation protocol.^30^ Hydrocortisone is one of the most commonly used corticosteroids in the management of septic shock patients due to its mineralocorticoid and glucocorticoid activity. Given these properties, hydrocortisone stimulates the release of aldosterone, promoting the reabsorption of salt and water and enhancing the vascular contractile response to pressor agents, thereby increasing blood pressure in septic shock patients. However, its use may introduce potential confounding in the measurement of MAP. Therefore, future randomized controlled trials are necessary to determine whether the combined administration of corticosteroids and methylene blue would positively influence MAP and to assess the extent of their respective effects.

Quantitative analysis of the three studies reporting mortality demonstrated a statistically significant reduction in the overall mortality rate among patients receiving methylene blue.^30^^,^^31^^,^^33^ However, the total sample size (n = 205) was very small. Hence, it is unpowered to detect any clinically meaningful outcomes for mortality rate and the pooled findings should be interpreted with caution. Future adequately powered studies are required to examine the impact of methylene blue on the mortality rate in septic shock patients. Serum lactate has been included in the recent definition of septic shock as it is considered an important biomarker of cellular stress in a refractory hypotension.^1^ Current guidelines recommend using lactate levels as biochemical indicators to guide resuscitation in early phases of septic shock.^3^ Our finding demonstrated that methylene blue was associated with significantly lower serum lactate levels.^30^^,^^31^^,^^33^ This suggests that methylene blue may exert favourable effects on tissue perfusion and anaerobic metabolism.^41^, ^42^, ^43^ However, the interpretation of lactate levels should be approached cautiously and considered alongside other hemodynamic parameters.

The time factor remains a critical element in the management of septic shock and early intervention is crucial to improving outcome.^3^ In our included studies, the timing of methylene blue administration and vasopressor titration varied across all the included studies. The majority of studies administered methylene blue within 24 hours after the initiation of vasopressors.^29^^,^^31^, ^32^, ^33^ It is hard to determine if there are possible delays between the onset of septic shock and the administration of methylene blue. It has been proposed that there is a ‘window of opportunity’ and time-sensitive for methylene blue to restore vascular resistance in septic shock effectively.^44^ A sepsis model in mice demonstrated that there exists a dynamic guanylate cyclase activity in three 8-hour windows.^44^ An increase in guanylate cyclase and iNOS is observed in the first 8-hour window, followed by an absence of guanylate cyclase expression and down-regulation of iNOS in the second 8-hour window.^45^ Then, there is an upregulation of guanylate cyclase and iNOS in the third 8-hour window.^45^ This suggests that the use of methylene blue as a last rescue therapeutic option may not act (second window) or act too late (third window) when the shock is irreversible with profound tissue hypoxia and intractable metabolic acidosis.^46^ In the current literature, methylene blue has been administered as a late rescue treatment during septic shock, and the impact of earlier administration as an adjunct is yet to be established.

There are some concerns that the use of methylene blue causes ventilation-perfusion mismatch and vasoconstriction via the inhibition of cGMP.^47^ Previous studies demonstrated a significant reduction in the PaO_2_/FiO_2_ ratio in the methylene blue group.^15^^,^^36^ Weingartner and colleagues specifically reported a decrease in the PaO_2_/FiO_2_ ratio from 168 (131‒215) mmHg to 132 (109‒156) mmHg following methylene blue administration (p < 0.05).^35^ However, it is worth noting that all the included patients in this study had acute lung injury, and seven of the ten included patients fulfilled the criteria for ARDS.^35^ Hence, the observed respiratory dysfunction may be attributed to septic shock secondary to respiratory pathology rather than the adverse effect of methylene blue. Our pooled results support this hypothesis by showing that methylene blue increases PaO_2_/FiO_2_ compared to the control group.^29^^,^^30^ Methylene blue is a safe drug when used in therapeutic doses of < 2 mg.kg^-1^.^38^ All our included studies used the dose of methylene blue < 2 mg.kg^-1^ and no significant adverse events were reported. There was an increase in the level of methaemoglobin in one of the included studies albeit not clinically significant.^29^ Other common side effects include blue skin and urine discoloration that are transient and benign.^38^ Clinicians should also be conscientious of the interference in pulse oximeter readings caused by methylene blue as it absorbs most light emitted by the pulse oximeter.^47^ This may be interpreted as a false reduction in circulating oxyhaemoglobin and reduced oxygen saturation.^48^ Furthermore, a study by Juffermans et al. demonstrated that high doses (> 7 mg.kg^-1^) of methylene blue may compromise splanchnic perfusion despite global haemodynamic enhancement.^39^ The usage of methylene blue is contraindicated in patients with Glucose-6-Phosphate Dehydrogenase (G6PD) deficiency as it increases the risk of haemolytic anaemia.^49^ Moreover, methylene blue also inhibits the monoamine oxidase enzyme, which may result in the manifestation of serotonin syndrome when used with monoamine oxidase inhibitors, such as antidepressant medications.^50^

Catecholamine-resistant shocked patients with high renin levels may benefit from early commencement of angiotensin II.^51^ Measurement of the metabolites of the nitric oxide pathway in the form of nitrite and nitrate may suggest early initiation of methylene blue. Markers to predict specific vasopressor responsiveness may be the ideal approach to managing hypotension in septic shock. Physiologically, diastolic arterial pressure and dynamic arterial elastance may be useful parameters to guide patients who will benefit from early vasopressors.^52^^,^^53^ Analysis of the kinetics of norepinephrine dose increment and the response to vasopressin treatment may suggest the need for early initiation of other vasopressors with different mechanisms of action.^54^ According to Kram et al., who studied patients with vasoplegic syndrome, the minimum methylene blue required to reduce norepinephrine needs was 1.4 mg.kg^-1^.^55^ For each incremental dose increase of 0.5 mg.kg^-1^, there is a 2.25 mg.kg^-1^ decrease in norepinephrine dosage.^55^ However, we have to be careful with the dose of methylene blue due to its side effects such as blue skin and urine discolouration.^38^ Hence, future studies on the pharmacokinetics and pharmacodynamics of methylene blue are warranted to gauge the optimal dose of methylene blue.

Our study has several limitations. Variations in methylene blue administration regimens and vasopressors titration protocols may have contributed to the substantial heterogeneity observed in our primary outcome. Additionally, all the included studies had small sample size and were underpowered to detect the primary outcome of MAP, as well as secondary outcomes such as mortality rate and other clinical parameters. The lack of blinding of participants and outcome assessors (researchers) introduces a risk of overestimating the treatment effects of methylene blue. Study participants and clinicians, aware of the intervention, may have influenced subjective outcomes such as hospital discharge timing, potentially biasing the length of hospital stay. Furthermore, the follow-up periods were short and heterogeneous across most RCTs, increasing the likelihood of underreporting adverse outcomes. Another limitation is that the causes of septic shock were not consistently reported across the included studies, which could introduce bias to our findings. The transient urine discoloration commonly associated with methylene blue administration may have unintentionally unblind clinicians, further increasing the risk of bias. None of the studies measured nitrite or nitrate levels, making it unclear whether the observed effects of MAP could be directly attributed to methylene blue. Finally, there is a risk of publication bias due to the limited number of studies published on the effects of methylene blue in septic shock patients. These limitations highlight the need for more robust, adequately powered, and methodologically sound randomized controlled trials to better assess the efficacy and safety of methylene blue in this context.

This meta-analysis demonstrated that methylene blue shows promise in the management of patients with septic shock. However, due to the limited sample size and the low quality of evidence in the included studies, its use requires further validation through larger, well-designed clinical trials. Future adequately powered randomized controlled trials are necessary to determine the optimal dosing and timing of methylene blue, as well as its effect on hemodynamic and clinical outcomes, before its routine implementation in clinical practice.

## Human ethics and consent to participate declarations

None.

## Previous presentation in conferences

RCoA Anaesthesia Research.

## Awards

RCoA Anaesthesia Research Presentation Award.

## Conflicts of interest and Funding

No potential conflict of interest relevant to this article was reported. No funding was received in support of this project.

## Appendix Supplementary materials

Image, application 1

## Footnotes

Supplementary material associated with this article can be found in the online version at doi:10.1016/j.bjane.2024.844580.
