# Supplementary material for: The use of methylene blue in adult patients with septic shock: a systematic review and meta-analysis
Source: Braz J Anesthesiol. 2024 Nov 29;75(1):844580. doi: 10.1016/j.bjane.2024.844580 (PMC11754512; doi:10.1016/j.bjane.2024.844580)
Supplement: Supplementary file 1 [file mmc1.docx]

**BJAN-D-24-00352_Supplementary Material**

**Supplemental Table 1** Definition of septic shock

| **Author** | **Year** | **Definition** |
| --- | --- | --- |
| Arzapalo | 2016 | Syndrome of systemic inflammatory response caused by an infectious agent; it presents with hypotension measured in millimetres of mercury that is refractory to hydric management, requiring the use of vasopressors measured in micrograms per kilogram of weight |
| Kirov | 2001 | Severe sepsis associated with MAP of %70 mmHg for at least 30 mins despite ﬂuid resuscitation, or with requirement for infusion of dopamine > 5 microgram.kg^-1^.min^-1^ and/or norepinephrine > 0.05 microgram.kg^-1^.min^-1^ and/or epinephrine > 0.05 microgram.kg^-1^.min^-1^ for at least 30 mins to maintain MAP between 70- and 90-mmHg |
| Lu | 2018 | ‒ |
| Lu | 2019 | MAP < 65 mmHg and serum lactate > 2 mmol.L^-1^ |
| Ibarra-Estrada | 2023 | Sepsis-3 criteria (highly suspected or confirmed infection, requiring norepinephrine to maintain a MAP ≥ 65 mmHg, and serum lactate > 2 mmol.L^-1^ after adequate fluid resuscitation) |

**Supplemental Table 2** Search Strategy.

| **Search Strategy for EMBASE databases (1974 to March 2023)** | |
| --- | --- |
| **Steps** | **Search String** |
| 1 | Methylene blue.mp. or exp methylene blue/31569 |
| 2 | Septic shock.mp. or exp septic shock/78426 |
| 3 | Exp hypovolemic shock/ or exp lipopolysaccharide-induced septic shock/ or exp endotoxic shock/ or shock.mp. or exp dengue shock syndrome/ or exp hemorrhagic shock/ or exp traumatic shock/ or exp vasodilatory shock/ or exp burn shock/ or exp cardiogenic shock/ or shock/ or exp septic shock/391264 |
| 4 | 2 or 3 / 391264 |
| 5 | 1 and 4 785 |
| 6 | limit 5 to human 632 |
| **Search Strategy for MEDLINE databases (1946 to March 2023)** | |
| **Steps** | **Search String** |
| 1 | Methylene blue.mp. or exp methylene blue/24793 |
| 2 | Septic shock.mp. or exp septic shock/40157 |
| 3 | Exp hypovolemic shock/ or exp lipopolysaccharide-induced septic shock/ or exp endotoxic shock/ or shock.mp. or exp dengue shock syndrome/ or exp hemorrhagic shock/ or exp traumatic shock/ or exp vasodilatory shock/ or exp burn shock/ or exp cardiogenic shock/ or shock/ or exp septic shock/278535 |
| 4 | 2 or 3 / 278535 |
| 5 | 1 and 4 363 |
| 6 | limit 5 to human 225 |
| **Search Strategy for CENTRAL databases (1946 to March 2023)** | |
| **Steps** | **Search String** |
| 1 | Methylene blue.mp. or exp methylene blue/ |
| 2 | Septic shock.mp. or exp septic shock/ |
| 3 | 1 and 2 / 23 |

**Supplemental Table 3** Characteristics of excluded studies.

| **Author**  **S3: Characteristics of Excluded Studies** | **Year** | **Design** | **Reason for exclusion** | **Country** | **n** |
| --- | --- | --- | --- | --- | --- |
| Andresen | 1998 | Case Series | Wrong Design | Chile | 10 |
| Carillo-Esper | 2010 | Case Report | Wrong Design | Mexico | 1 |
| Gachot | 1995 | Case Series | Wrong Design | France | 6 |
| Delgadillo | 2023 | Abstract for Conference | Wrong Design | Mexico | 45 |
| Grillo | 2018 | Abstract for Conference | Wrong Design | Spain | 50 |
| Heemskerk | 2008 | Case Series | Wrong Design | Netherlands | 9 |
| Schneider | 1992 | Case Report | Wrong Design | France | 2 |
| Naoum | 2022 | Retrospective Review | Wrong Design | USA | 223 |
| Brown | 1996 | Case Report | Wrong Design | Canada | 1 |
| Preiser | 1995 | Prospective, observational | Wrong design (Lack of Control Group) | Belgium | 14 |
| Donati | 2002 | Prospective, observational | Wrong design (lack of Control Group) | Belgium | 15 |
| Daeman- Gubbles | 1995 | Prospective, observational | Wrong design (Lack of Control Group) | Netherlands | 9 |
| Weigartner | 1999 | Prospective, observational | Wrong design (Lack of Control Group) | Brazil | 10 |
| Dumbarton | 2011 | Case Report | Wrong Design | Canada | 1 |
| Ismail | 2022 | RCT | Wrong Population (Neonate Population) | Egypt | 30 |
| Bitterman | 2020 | Retrospective Review | Wrong Population/ Design | Israel | 7 |
| Park BK | 2005 | Prospective, observational | Wrong design (Lack of Control Group) | Korea | 20 |
| Juffermans | 2010 | RCT | Wrong Design | Netherlands | 15 |
| Xu | 2021 | Narrative Review | Wrong Design | China | N/A |
| Li | 2014 | Observational Study | Wrong Design | China | 86 |
| Memis | 2002 | RCT | Wrong Design | Turkey | 30 |

**Supplemental Table 4** Characteristics of ongoing studies.

| **Author** | **Location** | **Status** | **Title** | **Comparator** | **Total Sample Size** | **Clinical Trial Number** |
| --- | --- | --- | --- | --- | --- | --- |
| Liu | China | Pending | Efficacy and Safety of Methylene Blue in the Treatment of Refractory Septic Shock: a Multicenter, Randomized, Placebo-Controlled Clinical Trial | Methylene Blue Group vs. Placebo Group | 100 | ChiCTR2300069430 |

**Supplemental Table 5** Risk of Bias Assessment of all the included studies

| **Author** | **Year** | **Overall Risk of Bias** | **Sequence Generation** | **Allocation Concealment** | **Blinding of Participants and Personnel** | **Blinding of outcome assessment** | **Incomplete outcome data** | **Selective outcome reporting** | **Other sources of Bias** |
| --- | --- | --- | --- | --- | --- | --- | --- | --- | --- |
| Ibarra-Estrada | 2023 | Low | Low | Low | Low | Low | Low | Low | Low |
| Arzapalo | 2016 | Low | Low | Low | Low | Low | Low | Low | Unknown |
| Kirov | 2001 | Unclear | Low | Low | Unknown | Unknown | Low | Low | Unknown |
| Lu | 2019 | High | Low | Unclear | High | Unclear | Low | Low | Unknown |
| Lu | 2018 | High | Low | Unclear | High | Unclear | Low | Low | Unknown |

**Supplemental Table 6** PRISMA checklist.

| **Section and Topic** | **Item #** | **Checklist item** | **Location where item is reported** |
| --- | --- | --- | --- |
| **TITLE** | | |  |
| Title | 1 | Identify the report as a systematic review. | 1 |
| **ABSTRACT** | | |  |
| Abstract | 2 | See the PRISMA 2020 for Abstracts checklist. | 1 |
| **INTRODUCTION** | | |  |
| Rationale | 3 | Describe the rationale for the review in the context of existing knowledge. | 2,3 |
| Objectives | 4 | Provide an explicit statement of the objective(s) or question(s) the review addresses. | 3 |
| **METHODS** | | |  |
| Eligibility criteria | 5 | Specify the inclusion and exclusion criteria for the review and how studies were grouped for the syntheses. | 4 |
| Information sources | 6 | Specify all databases, registers, websites, organisations, reference lists and other sources searched or consulted to identify studies. Specify the date when each source was last searched or consulted. | 4 |
| Search strategy | 7 | Present the full search strategies for all databases, registers and websites, including any filters and limits used. | 4 |
| Selection process | 8 | Specify the methods used to decide whether a study met the inclusion criteria of the review, including how many reviewers screened each record and each report retrieved, whether they worked independently, and if applicable, details of automation tools used in the process. | 4,5 |
| Data collection process | 9 | Specify the methods used to collect data from reports, including how many reviewers collected data from each report, whether they worked independently, any processes for obtaining or confirming data from study investigators, and if applicable, details of automation tools used in the process. | 4,5 |
| Data items | 10a | List and define all outcomes for which data were sought. Specify whether all results that were compatible with each outcome domain in each study were sought (e.g. for all measures, time points, analyses), and if not, the methods used to decide which results to collect. | 4,5 |
|  | 10b | List and define all other variables for which data were sought (e.g. participant and intervention characteristics, funding sources). Describe any assumptions made about any missing or unclear information. | 4,5 |
| Study risk of bias assessment | 11 | Specify the methods used to assess risk of bias in the included studies, including details of the tool(s) used, how many reviewers assessed each study and whether they worked independently, and if applicable, details of automation tools used in the process. | 5 |
| Effect measures | 12 | Specify for each outcome the effect measure(s) (e.g. risk ratio, mean difference) used in the synthesis or presentation of results. | 5 |
| Synthesis methods | 13a | Describe the processes used to decide which studies were eligible for each synthesis (e.g. tabulating the study intervention characteristics and comparing against the planned groups for each synthesis (item #5)). | 5 |
|  | 13b | Describe any methods required to prepare the data for presentation or synthesis, such as handling of missing summary statistics, or data conversions. | 5 |
|  | 13c | Describe any methods used to tabulate or visually display results of individual studies and syntheses. | 4,5 |
|  | 13d | Describe any methods used to synthesize results and provide a rationale for the choice(s). If meta-analysis was performed, describe the model(s), method(s) to identify the presence and extent of statistical heterogeneity, and software package(s) used. | 5 |
|  | 13e | Describe any methods used to explore possible causes of heterogeneity among study results (e.g. subgroup analysis, meta-regression). | 5 |
|  | 13f | Describe any sensitivity analyses conducted to assess robustness of the synthesized results. | N/A |
| Reporting bias assessment | 14 | Describe any methods used to assess risk of bias due to missing results in a synthesis (arising from reporting biases). | 5 |
| Certainty assessment | 15 | Describe any methods used to assess certainty (or confidence) in the body of evidence for an outcome. | 5 |
| **RESULTS** | | |  |
| Study selection | 16a | Describe the results of the search and selection process, from the number of records identified in the search to the number of studies included in the review, ideally using a flow diagram. | 6 |
|  | 16b | Cite studies that might appear to meet the inclusion criteria, but which were excluded, and explain why they were excluded. | 6 |
| Study characteristics | 17 | Cite each included study and present its characteristics. | 6 |
| Risk of bias in studies | 18 | Present assessments of risk of bias for each included study. | 6 |
| Results of individual studies | 19 | For all outcomes, present, for each study: (a) summary statistics for each group (where appropriate) and (b) an effect estimate and its precision (e.g. confidence/credible interval), ideally using structured tables or plots. | 6,7 |
| Results of syntheses | 20a | For each synthesis, briefly summarise the characteristics and risk of bias among contributing studies. | 6,7 |
|  | 20b | Present results of all statistical syntheses conducted. If meta-analysis was done, present for each the summary estimate and its precision (e.g. confidence/credible interval) and measures of statistical heterogeneity. If comparing groups, describe the direction of the effect. | 6,7 |
|  | 20c | Present results of all investigations of possible causes of heterogeneity among study results. | 6,7 |
|  | 20d | Present results of all sensitivity analyses conducted to assess the robustness of the synthesized results. | N/A |
| Reporting biases | 21 | Present assessments of risk of bias due to missing results (arising from reporting biases) for each synthesis assessed. | 6,7 |
| Certainty of evidence | 22 | Present assessments of certainty (or confidence) in the body of evidence for each outcome assessed. | 6,7 |
| **DISCUSSION** | | |  |
| Discussion | 23a | Provide a general interpretation of the results in the context of other evidence. | 8 |
|  | 23b | Discuss any limitations of the evidence included in the review. | 8,9,10,11 |
|  | 23c | Discuss any limitations of the review processes used. | 8,9,10,11 |
|  | 23d | Discuss implications of the results for practice, policy, and future research. | 11 |
| **OTHER INFORMATION** | | |  |
| Registration and protocol | 24a | Provide registration information for the review, including register name and registration number, or state that the review was not registered. | 4 |
|  | 24b | Indicate where the review protocol can be accessed, or state that a protocol was not prepared. | 4 |
|  | 24c | Describe and explain any amendments to information provided at registration or in the protocol. | N/A |
| Support | 25 | Describe sources of financial or non-financial support for the review, and the role of the funders or sponsors in the review. | 11 |
| Competing interests | 26 | Declare any competing interests of review authors. | 11 |
| Availability of data, code and other materials | 27 | Report which of the following are publicly available and where they can be found: template data collection forms; data extracted from included studies; data used for all analyses; analytic code; any other materials used in the review. | 4,6 |

**Supplementary Figure 1** Forest plot of mean arterial pressure (mmHg). Subgroup analysis methylene blue significantly reduced mean arterial pressure in comparison to the control group in the low risk of bias subgroup.


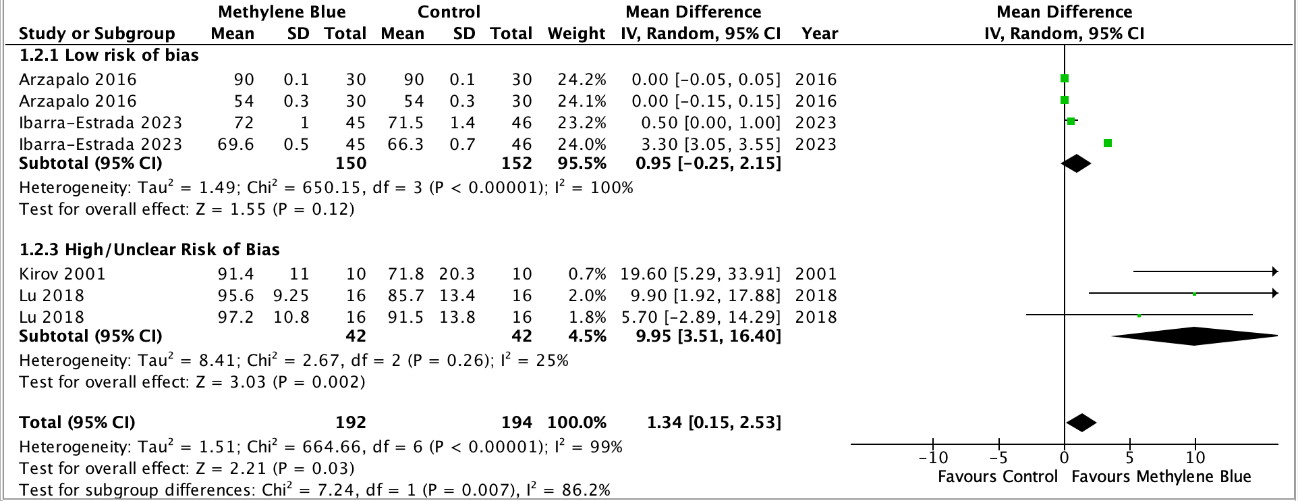


**Supplementary Figure 2** Forest plot of mortality rate. Methylene blue group is associated with significant reduction in mortality rate compared to the control group.


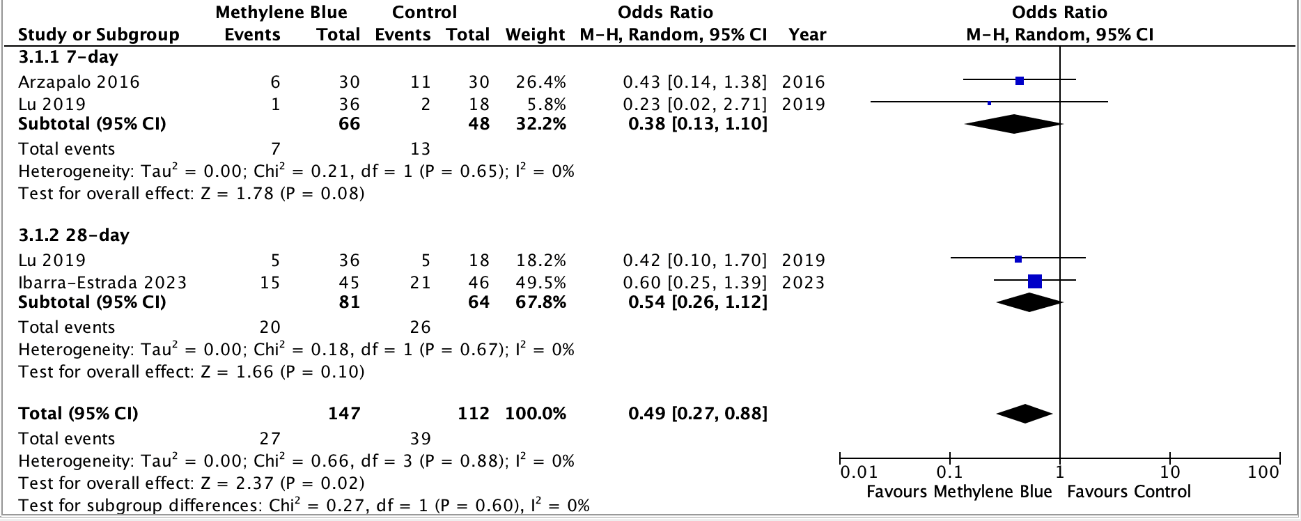


**Supplementary Figure 3** Forest plot of serum lactate (mmoL.L^-1^). Methylene blue is associated with significant reduction of serum lactate.


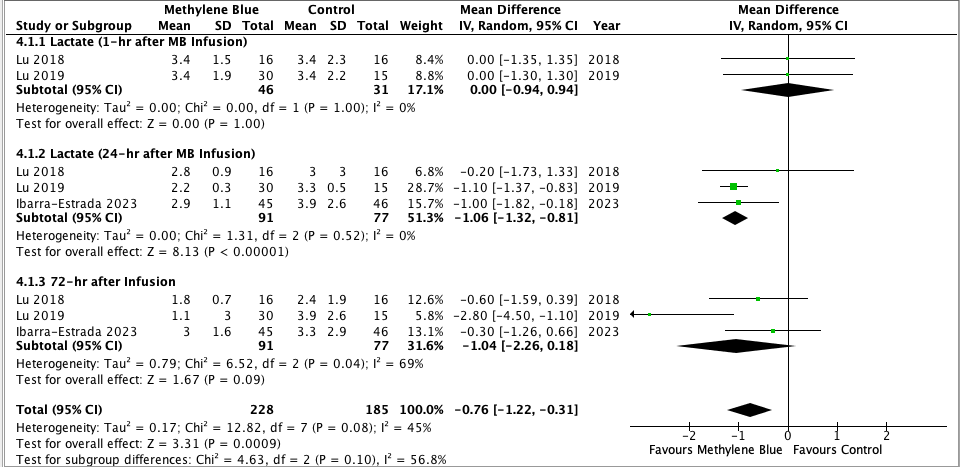


**Supplementary Figure 4** Forest Plot of PaO_2_/FiO_2._ Methylene blue is co-related with a significant increase in PaO_2_/FiO_2_ compared to the control group.


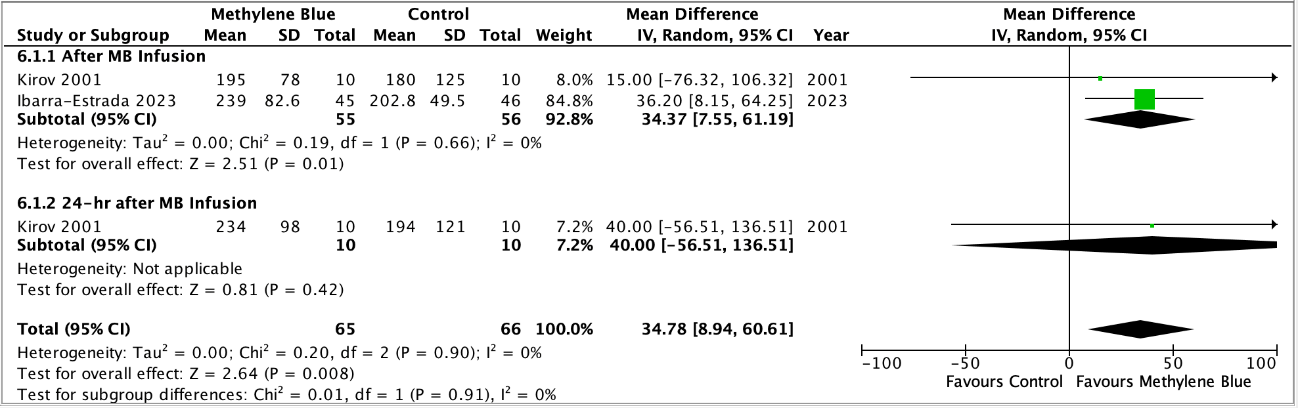


**Supplementary Figure 5** Forest plot of length of hospital stay (days). Methylene blue significantly reduced the duration of hospital stay in comparison to the control group.


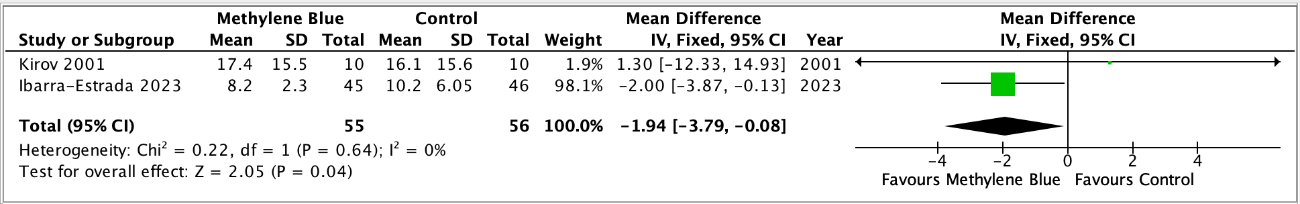


**Supplementary Figure 6** Forest plot of heart rate_._ There is no significant difference between methylene blue and the control group on the heart rate across different time points after infusion of methylene blue.


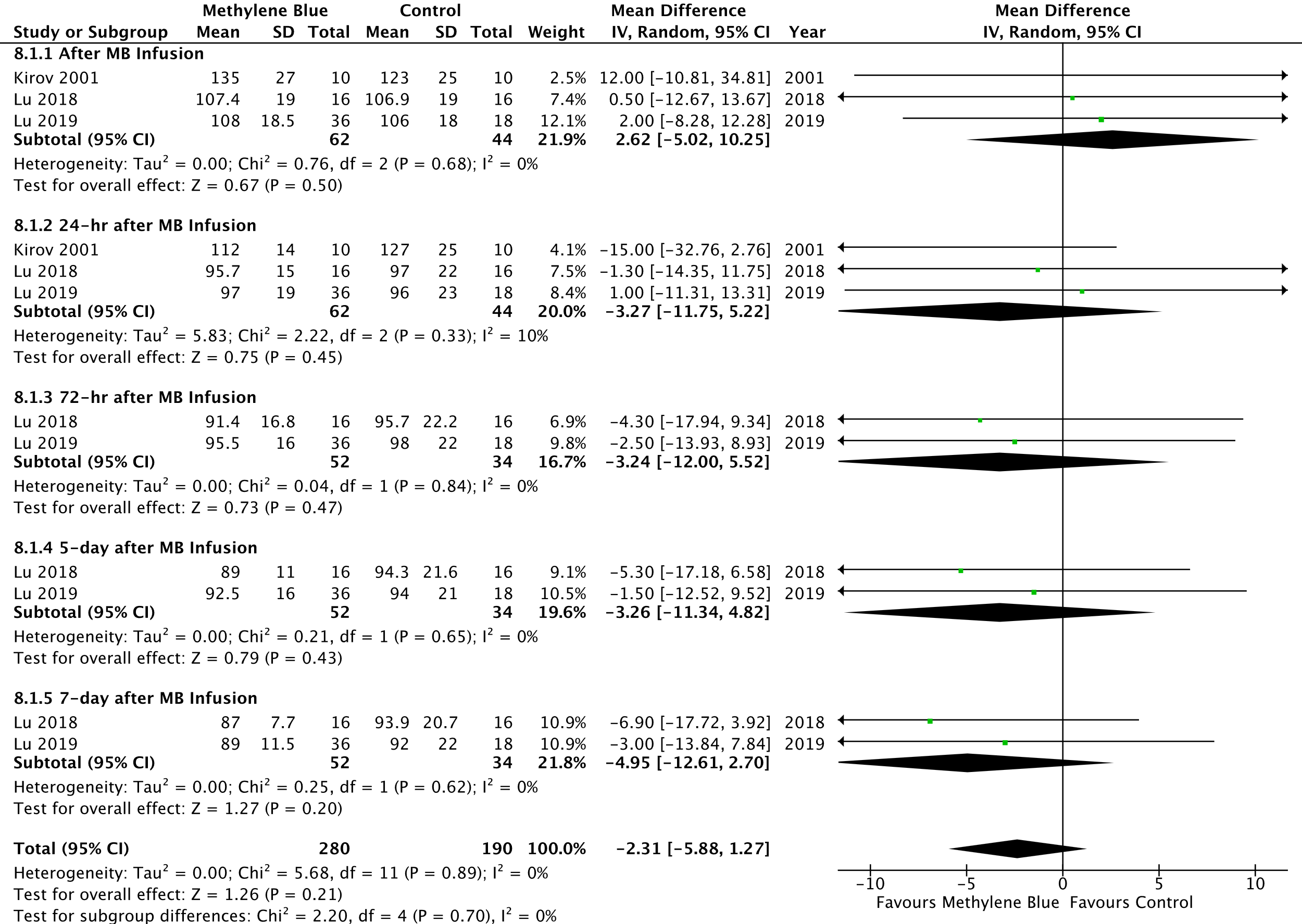


**Data availability statement**

All data on the primary outcome and secondary outcomes are included within this paper and its Supplementary Information files.
